# Supplementary material for: Multi‐dataset identification of innovative feature genes and molecular mechanisms in keratoconus
Source: J Cell Mol Med. 2024 Sep 19;28(18):e70079. doi: 10.1111/jcmm.70079 (PMC11412914; doi:10.1111/jcmm.70079)
Supplement: Supplementary file 2 — Material S2. [file JCMM-28-e70079-s002.zip › 7.GSEA analysis/3.DBNDD1/KEGG_PANCREATIC_CANCER.html]

Details for gene set KEGG\_PANCREATIC\_CANCER[GSEA]

|  || Dataset | DBNDD1.group.cls#h\_versus\_l |
| Phenotype | group.cls#h\_versus\_l |
| Upregulated in class | h |
| GeneSet | KEGG\_PANCREATIC\_CANCER |
| Enrichment Score (ES) | 0.1778401 |
| Normalized Enrichment Score (NES) | 0.78971636 |
| Nominal p-value | 0.72912425 |
| FDR q-value | 1.0 |
| FWER p-Value | 1.0 |
Table: GSEA Results Summary

  

Fig 1: Enrichment plot: KEGG\_PANCREATIC\_CANCER      
 Profile of the Running ES Score & Positions of GeneSet Members on the Rank Ordered List

  

| SYMBOL | TITLE | RANK IN GENE LIST | RANK METRIC SCORE | RUNNING ES | CORE ENRICHMENT || 1 | PIK3R5 | na | 35 | 0.708 | 0.1170 | Yes |
| 2 | ARHGEF6 | na | 1201 | 0.362 | 0.1438 | Yes |
| 3 | RAC3 | na | 1824 | 0.312 | 0.1778 | Yes |
| 4 | TGFBR2 | na | 4515 | 0.197 | 0.1330 | No |
| 5 | E2F2 | na | 6645 | 0.133 | 0.0938 | No |
| 6 | PIK3R1 | na | 6912 | 0.126 | 0.1071 | No |
| 7 | SMAD2 | na | 7383 | 0.116 | 0.1129 | No |
| 8 | AKT2 | na | 9388 | 0.080 | 0.0685 | No |
| 9 | CCND1 | na | 9676 | 0.076 | 0.0728 | No |
| 10 | MAPK3 | na | 9865 | 0.073 | 0.0796 | No |
| 11 | RB1 | na | 10147 | 0.069 | 0.0831 | No |
| 12 | MAPK10 | na | 10497 | 0.065 | 0.0838 | No |
| 13 | MAPK9 | na | 11537 | 0.052 | 0.0625 | No |
| 14 | PIK3R2 | na | 11699 | 0.050 | 0.0662 | No |
| 15 | SMAD4 | na | 11961 | 0.047 | 0.0665 | No |
| 16 | ERBB2 | na | 13132 | 0.037 | 0.0390 | No |
| 17 | TGFB3 | na | 13624 | 0.033 | 0.0303 | No |
| 18 | TP53 | na | 13962 | 0.030 | 0.0256 | No |
| 19 | KRAS | na | 14127 | 0.029 | 0.0258 | No |
| 20 | PIK3R3 | na | 14144 | 0.029 | 0.0301 | No |
| 21 | RALGDS | na | 14647 | 0.025 | 0.0199 | No |
| 22 | RAC1 | na | 14759 | 0.025 | 0.0207 | No |
| 23 | RALA | na | 15036 | 0.023 | 0.0165 | No |
| 24 | PLD1 | na | 15421 | 0.020 | 0.0088 | No |
| 25 | BRAF | na | 15471 | 0.019 | 0.0106 | No |
| 26 | PIK3CA | na | 17073 | 0.009 | -0.0341 | No |
| 27 | RALBP1 | na | 17251 | 0.008 | -0.0379 | No |
| 28 | VEGFB | na | 17569 | 0.006 | -0.0461 | No |
| 29 | PIK3CB | na | 18338 | 0.000 | -0.0682 | No |
| 30 | CHUK | na | 18953 | -0.004 | -0.0852 | No |
| 31 | RAF1 | na | 19005 | -0.005 | -0.0859 | No |
| 32 | AKT1 | na | 19066 | -0.005 | -0.0868 | No |
| 33 | TGFB2 | na | 19303 | -0.007 | -0.0925 | No |
| 34 | EGF | na | 19333 | -0.007 | -0.0921 | No |
| 35 | CASP9 | na | 19881 | -0.012 | -0.1059 | No |
| 36 | STAT1 | na | 19966 | -0.013 | -0.1062 | No |
| 37 | SMAD3 | na | 19999 | -0.013 | -0.1049 | No |
| 38 | RALB | na | 20318 | -0.016 | -0.1115 | No |
| 39 | BAD | na | 20453 | -0.017 | -0.1125 | No |
| 40 | RAC2 | na | 20865 | -0.021 | -0.1209 | No |
| 41 | CDK4 | na | 21131 | -0.023 | -0.1247 | No |
| 42 | CDKN2A | na | 21295 | -0.025 | -0.1253 | No |
| 43 | EGFR | na | 22041 | -0.033 | -0.1413 | No |
| 44 | IKBKB | na | 22300 | -0.035 | -0.1429 | No |
| 45 | MAPK8 | na | 22620 | -0.039 | -0.1457 | No |
| 46 | CDC42 | na | 22626 | -0.039 | -0.1394 | No |
| 47 | MAPK1 | na | 22705 | -0.040 | -0.1350 | No |
| 48 | TGFA | na | 22844 | -0.041 | -0.1321 | No |
| 49 | IKBKG | na | 23063 | -0.044 | -0.1311 | No |
| 50 | E2F3 | na | 24031 | -0.057 | -0.1495 | No |
| 51 | JAK1 | na | 24146 | -0.058 | -0.1431 | No |
| 52 | ARAF | na | 24817 | -0.068 | -0.1511 | No |
| 53 | PIK3CD | na | 24971 | -0.070 | -0.1438 | No |
| 54 | STAT3 | na | 25462 | -0.078 | -0.1449 | No |
| 55 | NFKB1 | na | 25842 | -0.084 | -0.1418 | No |
| 56 | TGFBR1 | na | 26390 | -0.095 | -0.1417 | No |
| 57 | BCL2L1 | na | 26569 | -0.099 | -0.1303 | No |
| 58 | CDK6 | na | 27414 | -0.117 | -0.1351 | No |
| 59 | PIK3CG | na | 27455 | -0.118 | -0.1166 | No |
| 60 | BRCA2 | na | 27586 | -0.121 | -0.1003 | No |
| 61 | E2F1 | na | 27800 | -0.126 | -0.0853 | No |
| 62 | RELA | na | 28616 | -0.147 | -0.0844 | No |
| 63 | MAP2K1 | na | 29246 | -0.164 | -0.0752 | No |
| 64 | PGF | na | 29370 | -0.168 | -0.0507 | No |
| 65 | RAD51 | na | 29952 | -0.187 | -0.0362 | No |
| 66 | VEGFA | na | 30038 | -0.189 | -0.0071 | No |
| 67 | TGFB1 | na | 30914 | -0.218 | 0.0040 | No |
| 68 | VEGFC | na | 31739 | -0.251 | 0.0221 | No |
| 69 | AKT3 | na | 33656 | -0.385 | 0.0309 | No |
Table: GSEA details [plain text format]

  

Fig 2: KEGG\_PANCREATIC\_CANCER      
 Blue-Pink O' Gram in the Space of the Analyzed GeneSet

  

Fig 3: KEGG\_PANCREATIC\_CANCER: Random ES distribution      
 Gene set null distribution of ES for **KEGG\_PANCREATIC\_CANCER**
